# Real-World Use of Molnupiravir in the Treatment of Outpatients with SARS-CoV-2 Infection—A Patient Profile Based on the Experience of a Tertiary Infectious Disease Center

**DOI:** 10.3390/ph15091065

**Published:** 2022-08-27

**Authors:** Anca Streinu-Cercel, Victor Daniel Miron, Alina Alexandra Oană, Mădălina Irimia, Ramona Ștefania Popescu, Ioana Andreea Dărămuș, Maria Magdalena Moțoi, Gabriela Jana Ceapraga, Oana Săndulescu

**Affiliations:** 1 Carol Davila University of Medicine and Pharmacy, 050474 Bucharest, Romania; 2 National Institute for Infectious Diseases “Prof. Dr. Matei Balș”, 021105 Bucharest, Romania

**Keywords:** COVID-19, SARS-CoV-2, molnupiravir, antiviral, real-world data

## Abstract

During the current pandemic, the gap between fundamental research and clinical practice has been narrowing at a faster pace than ever before. While clinical trials play the main role of confirming the safety and efficacy of new drugs, a drug’s introduction into clinical practice creates the need for further research in order to best position the use of the novel drug in terms of when, to whom, and how it would be best administered to achieve the best possible outcome under feasible clinical circumstances. We briefly present the results of a retrospective analysis of the characteristics of outpatients treated with molnupiravir in a tertiary care infectious disease hospital in Bucharest, Romania, between February and March 2022, when Romania was experiencing its fifth wave of COVID-19. A total of 46 outpatients received molnupiravir treatment and had complete clinical data available; of them, 56.5% (*n* = 20) were males and the median age was 48.5 years (IQR: 37.8, 67.0 years). A total of 54.2% (*n* = 26) of patients had at least one chronic condition. Of the 45 patients who underwent lung CT imaging evaluation, 13 (28.9%) showed changes suggestive of COVID-19 pneumonia. COVID-19 vaccination status was strongly protective for pneumonia (*p* = 0.002). All patients were symptomatic, and molnupiravir was initiated at a mean time from onset of symptoms of 3.5 (±1.5) days. At phone follow-up 5 days after the initial evaluation and initiation of molnupiravir treatment, all patients, except for one, confirmed a favorable course under treatment, with no worsening of COVID-19 severity and improvement in symptoms; none of them progressed to respiratory failure or required hospitalization. In conclusion, treatment was well tolerated and associated a favorable outcome of COVID-19 in routine practice in a clinical population that was slightly older and had a smaller burden of comorbidities and a higher rate of COVID-19 vaccination compared to that from the pivotal trial.

## 1. Introduction

The COVID-19 pandemic has been and still is a major challenge for the medical community. For more than two years, finding effective therapeutic targets against SARS-CoV-2 has become a priority for medical research. However, the virus seems to be “running faster” and undergoing structural changes that make it difficult to develop high-efficacy antivirals and highlight the importance of assessing the efficacy of the existing antivirals against each emerging variant of concern. During the current pandemic, the gap between fundamental research and clinical practice has been narrowing at a faster pace than ever before. While clinical trials play the main role of confirming the safety and efficacy of new drugs, the drug’s introduction into clinical practice creates the need for further research in order to: (a) see whether real-world use leads to comparable outcomes, (b) determine to which patient population the clinical trials results can be generalized or extended, and (c) best position the use of the novel drug in terms of when, to whom, and how it would be best administered in order to achieve the best possible outcome, under feasible clinical circumstances.

Despite intense and meaningful clinical research performed with immense effort during the first years of the pandemic, it is becoming increasingly clear that a one-size-fits-all approach to standard-of-care therapy is not appropriate for COVID-19. We have indeed refined clinical indicators [1], laboratory markers [2,3,4], and imaging techniques and scores [5] to aid in early recognition of patients at high risk of severe COVID-19 outcomes, but treatment is much less standardized.

In February 2022, a novel antiviral with oral administration, molnupiravir, became available in Romania for the treatment of outpatients with mild or moderate COVID-19, at risk of progression to severe disease, who ideally present for clinical assessment during the first 5 days since symptom onset. Molnupiravir has good oral bioavailability, and following absorption, it is cleaved in the plasma, distributed to tissues, and transformed into the active β-D-N4-hydroxycytidine triphosphate form [6]. This active form of the drug is then used as substrate by the viral-RNA-dependent RNA polymerase (RdRp), and the resulting RNA template induces the synthesis of mutated viral RNA, which is unable to lead to the formation of new SARS-CoV-2 virions, thus stopping viral replication [7,8,9]. According to its regulatory approval, molnupiravir should not be used during pregnancy and effective contraception should be used in women of childbearing potential for at least 4 days following completion of treatment, and in men for at least 3 months after treatment. Breastfeeding is also contraindicated, for 4 days after the last administered dose [10].

Through this brief report, we aimed to analyze data regarding the real-world clinical use of molnupiravir and to assess whether our patient profile and short-term COVID-19 outcomes are comparable to those of clinical trial participants from the pivotal MOVe-OUT trial [11].

## 2. Materials and Methods

The National Institute for Infectious Diseases (NIID) “Prof. Dr. Matei Balș” is the leading tertiary infectious diseases hospital in Romania, and from March 2020 through most of 2020, 2021, and early 2022 it has been fully dedicated to the care of patients with COVID-19. Due to the high number of COVID-19 cases, the hospital operates an outpatient evaluation center for patients with mild and moderate forms of SARS-CoV-2 infection. Here, patients receive treatment tailored to the form of the disease and the patient’s clinical profile according to the protocol of care for patients with COVID-19 in our country [12].

Confirmation of SARS-CoV-2 infection is done for each patient at the evaluation center by any EC-recognized test based on either nucleic acid amplification (NAAT), reverse transcription polymerase chain (RT-PCR) or rapid antigen identification from a nasopharyngeal and/or oropharyngeal sample [13].

All outpatients are evaluated clinically and with laboratory and/or imaging investigations at baseline and are prescribed COVID-19 treatment. Subsequently, they are monitored by physical or phone follow-up for at least 5 days after the initial evaluation to assess clinical improvement (i.e., remission of clinical signs and symptoms such as fever, chills, cough, dyspnea, sore throat, myalgia, fatigue, headache, nasal obstruction, nausea, and diarrhea) or clinical worsening (defined as transition to a higher disease severity compared to baseline, i.e., from mild to moderate or from moderate to severe).

Baseline disease severity is defined as: mild (symptomatic patients, without dyspnea, without pneumonia seen on chest CT or X-ray), moderate (symptomatic patients, with pneumonia seen on chest CT or X-ray), severe (symptomatic patients, with pneumonia, requiring oxygen supplementation with ambient air peripheral oxygen saturation ≤93% or respiratory rate ≥30/min), or critical (patients requiring management in the intensive care unit for COVID-19 with acute respiratory distress, sepsis, altered consciousness, and/or multiple organ dysfunction) [12].

We conducted a retrospective analysis among all patients evaluated in the outpatient department of NIID “Prof. Dr. Matei Balș”, Bucharest, Romania, between February and March 2022, when Romania was experiencing its fifth wave of COVID-19, dominated by omicron BA.1 and BA.2 [14]. We included in this analysis all consecutive patients who received molnupiravir treatment and were subsequently followed up at least 5 days after treatment initiation. We excluded from the analysis patients for whom complete clinical and/or laboratory data were not available, those who were lost to follow-up and those who also received another antiviral or monoclonal antibody therapy.

Data were analyzed using IBM SPSS Statistics for Windows, version 25 (IBM Corp., Armonk, NY, USA). Categorical variables were analyzed using the Chi-square test with risk calculation by odds ratio (OR) reported with 95% confidence interval (95% CI). The rates in two groups were assessed with the Z-score test for two population proportions. For continuous variables, after checking variable distribution, we report the mean, standard deviation (SD), and *t*-test results or median, interquartile range (IQR), and Mann Whitney-U test results.

## 3. Results

### 3.1. Molnupiravir—Data on Use in Our Clinical Practice

A total of 254 outpatients were evaluated by our team during the period under review (February–March 2022). Of these, 72 (28.3%) received treatment with molnupiravir, but in our analysis we included only 46 (18.1%) of the patients, i.e., those who met the inclusion criteria specified above. We identified an approximately equal sex distribution (56.5%, *n* = 26 males) and a median age of 48.5 years (IQR: 37.8, 67.0 years) (min. 20 years, and max. 85 years). There was no significant age difference by sex (48.5 years (IQR: 34.8, 53.8 years) for males, and 48.5 years (IQR: 38, 68 years) for females, *p* = 0.431).

The majority of patients (82.6%, *n* = 38) had received a full course of COVID-19 vaccination at least one month prior to the acute episode of illness, and 65.8% of patients had also received a complementary booster dose (Table 1). Vaccinated patients were significantly younger than unvaccinated patients (47.5 years (IQR: 35.5, 58.0) vs. 68 years (IQR: 45.0, 72.3), *p* = 0.030, r = 0.32).

A total of 54.2% (*n* = 26) of patients had at least one chronic condition. Obesity (30.4%, *n* = 14) and cardiovascular disease (28.3%, *n* = 13) were the most common (Table 1). There was no difference among sexes regarding the presence of chronic diseases (*p* = 0.264), but higher age correlated with having at least one chronic disease (53 years (IQR: 41.5, 68 years) vs. 45 years (IQR: 31.5, 52 years), *p* = 0.028, r = 0.32). Only 10.9% (*n* = 5) of patients were smokers.

All patients were symptomatic. In all patients included in the analysis, molnupiravir was initiated in the same day when they presented to our hospital for evaluation. The mean time from onset of symptoms to hospital presentation was 3.5 (±1.5) days. Cough (69.6%, *n* = 32), sore throat (54.3%, *n* = 25), and headache (52.2%, *n* = 24) were the most common presenting complaints. Headache was more common in females (70.0% vs. 38.5%, *p* = 0.034, χ^2^ = 4.5, OR = 0.3, 95%CI: 0.1–0.9), and vaccinated patients had lower odds of presenting systemic symptoms such as malaise (50. 0% vs. 15.8%, *p* = 0.033, χ^2^ = 4.5, OR = 0.2, 95%CI: 0.04–0.9) and myalgia (75.0% vs. 30.1%, *p* = 0.019, χ^2^ = 3.7, OR = 0.2, 95% CI: 0.02–0.8) (Table 2).

Laboratory investigations showed a number of findings, with 37.0% (*n* = 17) of patients showing decreased lymphocyte counts and 34.8% (*n* = 16) showing decreased platelet counts. Approximately two thirds (60.5%, *n* = 26) had inflammatory syndrome (increases in C-reactive protein above 5 mg/L). A percentage of 23.9% (*n* = 11) showed increases in aspartate aminotransferase (AST) and 26.1% (*n* = 12) in alanine aminotransferase (ALT). Table 3 shows the mean or median values of laboratory parameters and the frequency of patients with results outside normal ranges. We did not identify variations in laboratory parameters by sex or vaccination status (*p* > 0.05 in all cases).

With the exception of one patient, 45 (97.8%) underwent lung CT imaging evaluation. Of these, 13 (28.9%) showed changes suggestive of COVID-19 pneumonia. Patient sex was not associated with the presence of pneumonia (30.8% for males vs. 25.0% for females, *p* = 0.745), but vaccination status was strongly protective for pneumonia, thus 75.0% (*n* = 6) of the unvaccinated patients had lung changes on CT compared to 18.4% (*n* = 7) among vaccinated patients (*p* = 0.002, χ^2^ = 10.1, OR = 0.08, 95%CI: 0.01–0.47).

At phone follow-up 5 days after the initial evaluation and initiation of molnupiravir treatment, all patients, except for one, confirmed a favorable course under treatment, with no worsening of COVID-19 severity and improvement in symptoms; none of them progressed to respiratory failure or required hospitalization. The only patient who returned for re-evaluation at 3 days was an 85-year-old unvaccinated male, known to have controlled asthma, with mild interstitial pneumonia on CT at the initial evaluation, who returned for chest constriction and effort dyspnea, but did not require oxygen supplementation nor hospitalization. One other patient reported a maculo-papular rash that appeared on the third day of administration of molnupiravir and was not associated with any hypersensitivity signs. The patient was treated with antihistamine drugs, and the rash subsided after a total of 5 days.

### 3.2. Comparison of Real-World Data with Clinical Trial Data

We compared our clinical population with that from the interim analysis of the MOVe-OUT pivotal trial [11] that led to the initial emergency approval of molnupiravir by the regulating authorities. In our clinical practice, we had slightly more male patients (56.5% vs. 46.4%, *p* = 0.704, Z-score = −0.4), with higher ages (median of 48.5 vs. 42.0 years), and lower baseline C-reactive protein values (14.1 mg/L vs. 21.23 mg/L [15]). A smaller percentage of our patients had at least one chronic condition (54.2% vs. 99.4%, *p* < 0.001, Z-score = −16.2), with a lower prevalence of obesity (30.4% vs. 75.1%, *p* < 0.001, Z-score = −6.6), a comparable prevalence of diabetes mellitus (15.2% vs. 14.9%, *p* = 0.960, Z-score = 0.05), and a higher prevalence of significant cardiovascular disease (28.3% vs. 12.0%, *p* = 0.002, Z-score = 3.2) and active neoplasia (10.9% vs. 1.8%, *p* < 0.001, Z-score = 3.9). Compared to the MOVe-OUT trial, which specifically excluded vaccinated patients, 82.6% of our patients had been vaccinated against COVID-19, and a smaller percentage of them had moderate COVID-19 at baseline (28.9% vs. 44.0%, *p* = 0.037, Z-score = −2.1).

## 4. Discussion

In our routine clinical practice, treatment with molnupiravir appeared to be well tolerated, and it was associated with a favorable outcome of COVID-19 in a clinical population that was slightly older but had a smaller burden of comorbidities compared to that from the pivotal trial [11] and from clinical practice in Italy [16]. Furthermore, most of our patients had been vaccinated against COVID-19. To the best of our knowledge, this is one of the first reports on the use of molnupiravir in vaccinated patients, and our data come to confirm that clinical outcomes remain favorable, even more so, in this patient population as well, which is in line with data coming from real-world practice in Italy, where 89.1% of patients treated with molnupiravir had received at least one dose of a COVID-19 vaccine and 56.8% had been fully and recently vaccinated [16]. De Vito et al. have also shown that early treatment with molnupiravir, defined as treatment started within the first 3 days from symptom onset, decreased the risk of COVID-19 progression [16]. In our study, treatment was initiated at a mean duration of 3.5 days since onset, which lead to a high proportion of favorable outcomes.

Outcome data were not directly comparable between our patients and the MOVe-OUT pivotal trial participants, for a number of reasons. First, none of our patients died or progressed to requirement of hospitalization, which prevented a comparison with the MOVe-OUT 29-day endpoint of mortality or hospitalization [11]; this could in part be due to the fact that our population included a large share of previously vaccinated patients, who had overall lower rates of worse COVID-19 outcomes. Second, the MOVe-OUT trial was performed mainly during the delta wave, with most (33.1%) subjects confirmed with delta variant, and 44.6% without evaluable sequence data at the time of publication [11], and our real-world use occurred during the initial omicron wave, which started in late December 2021 [17], reached its peak in February 2022, and mainly subsided by the end of March 2022 in Romania.

In real-word medical practice, clinical decision-making is based on the best available evidence but also on a comprehensive evaluation of each individual patient [18]. Furthermore, a tendency for following the “tried and true” path can be seen, whereby medical decisions are based on past treatment experiences. However, during a pandemic that is due to a novel virus, the “tried and true” path is only now beginning to form as new treatment options become available. Therefore, there is the need to better understand and position each novel drug.

Whenever a new drug reaches the clinic, there can be two main approaches to prescribing practices: some practitioners will immediately start embracing the new drug and will base their prescription on the existing evidence, initially coming from clinical trials, and subsequently, from real-world data, while others will remain generally cautious and will “watch and wait” until more clinical experience is gathered from their immediate colleagues from the same clinical practice [19,20]. In both of these scenarios, information coming from real-world medical practice is needed. In the case of early prescribers, it will help adapt and better calibrate their prescribing practice, and in the case of late prescribers, it might help enlarge their prescribing portfolio and build confidence around the use of newly approved drugs.

Clinical decision support systems are available for antibiotic prescription [21,22], but much work is still needed to refine antiviral prescribing practices. While antiviral treatment of chronic infections such as HIV or hepatitis B or C is mostly standardized, less data is available for antiviral treatment of acute viral infections. For example, for influenza, despite the availability of efficient antivirals for more than 10 years, controversies in prescribing practices still exist, rates of prescriptions ranging from 15% in outpatients [23] to 55.2% [24] in hospitalized patients. Furthermore, recent clinical observations highlight these low prescribing rates and emphasize the importance of administering influenza antivirals to specific patient populations even if they present after the first 48 h since symptom onset [24].

Having learned important epidemiological lessons from influenza [25], it is important to be able to translate these findings into actionable clinical advice and to see to what extent we can extrapolate them to COVID-19. Therefore, now that antivirals against SARS-CoV-2 are becoming available, our main focus should be on identifying the patient population who would most benefit from treatment, ensuring timely access to treatment, and tailoring treatment to the patient’s needs. In Romania, four antivirals against SARS-CoV-2 are currently recommended by the national treatment protocol, i.e., the intravenously-administered remdesivir and the orally-administered molnupiravir, nirmatrelvir/r, and repurposed influenza antiviral favipiravir [12]. However, nirmatrelvir/r is not yet available in clinical practice in Romania and data on favipiravir is still largely inconclusive, with clinical trials and meta-analyses suggesting some effect on faster viral clearance and clinical improvement in hospitalized patients but no significant impact on mortality or in outpatients [26,27]. Molnupiravir thus remains the only option available for oral outpatient treatment, which may lead to difficult clinical decisions when the patient’s profile does not exactly match the on-label indications but no other options are available, either because intravenous administration might not be feasible or acceptable to the patient, or because of temporary unavailability in the supply of other drugs, which is an issue that has come up repeatedly during the current pandemic.

Therefore, to better refine the currently available treatment protocols, there is an important need for continued development of further clinical options, coupled with better and continuous supply of the existing drugs, and publication of real-word data analyses, in order to refine therapeutic decisions for patients with COVID-19.

Our study has a number of limitations. Specifically, it is a single-center study with a relatively small sample size. Furthermore, the duration of follow-up is short, which is a known limitation of clinical data based on real-world outpatient management. However, it also has a set of strengths: the study site is one of the main reference centers in Romania, which, taken together with the fact that our clinical population had a relatively low rate of missing data, allows, to a certain extent, a better generalizability of the data to the wider patient population attending COVID-19 outpatient care in our setting.

## 5. Conclusions

In conclusion, we have presented the real-world profile of outpatients treated with molnupiravir in a specialty reference hospital for infectious diseases. Treatment was well tolerated and associated a favorable outcome of COVID-19 in our routine practice in a clinical population that was slightly older and had a smaller burden of comorbidities and a higher rate of COVID-19 vaccination compared to that from the pivotal trial. Our data fill a gap in current knowledge regarding the actual use of novel drugs that reach clinical practice.

## Figures and Tables

**Table 1:** Distribution of chronic conditions in patients treated with molnupiravir.

Characteristics	Number	Percentage (%)
**Demographic data**		
Male sex	26	56.5
Female sex	20	43.5
Median age (years)	48.5 (IQR: 37.8, 67.0)	
**Chronic condition**		
Obesity	14	30.4
Cardiovascular disease	13	28.3
Asthma	2	4.3
COPD	1	2.2
Neoplasia	5	10.9
Chronic kidney disease	0	0.0
Autoimmune disease	1	2.2
Diabetes mellitus	7	15.2
Other	3	6.5
1 chronic condition	15	60.0 *
2 chronic conditions	7	28.0 *
3 chronic conditions	3	12.0 *
**COVID-19 vaccination status**		
2 doses mRNA vaccine	11	29.0
3 doses mRNA vaccine	25	65.8
1 dose viral vector vaccine **	1	2.6
2 doses viral vector vaccine	1	2.6

COPD—chronic obstructive pulmonary disease. * Percentage calculated from the total number of patients with at least one chronic condition. ** This patient was considered fully vaccinated, having received a viral vector vaccine approved for single-dose administration.

**Table 2:** Analysis of the presenting complaints according to sex and vaccination status.

Symptoms	Male (*N* = 26)	Female (*N* = 20)	*p*-Value	Vaccinated (*N* = 36)	Unvaccinated (*N* = 8)	*p*-Value	Total (*N* = 46)
Cough	15 (57.7)	17 (85.0)	0.046	25 (65.8)	7 (87.5)	0.225	32 (69.6)
Headache	10 (38.5)	14 (70.0)	0.034	19 (52.8)	5 (62.5)	0.520	24 (52.2)
Sore throat	13 (50.0)	12 (60.0)	0.500	23 (63.9)	2 (25.0)	0.067	25 (54.3)
Myalgia	9 (34.6)	8 (40.0)	0.708	11 (30.1)	6 (75.0)	0.019	17 (37.0)
Fever	9 (34.6)	8 (40.0)	0.708	13 (34.2)	4 (50.0)	0.400	17 (37.0)
Rhinorrhea	8 (30.8)	9 (45.0)	0.322	13 (34.2)	4 (50.0)	0.400	17 (37.0)
Malaise	5 (19.2)	5 (25.0)	0.638	6 (15.8)	4 (50.0)	0.033	10 (21.7)
Chest pain	2 (7.7)	1 (5.0)	NA	1 (2.8)	2 (25.0)	NA	3 (6.5)
Diarrhea	2 (7.7)	0 (0.0)	NA	0 (0.0)	2 (25.0)	NA	2 (4.3)

NA—not applicable. Data is presented as number (percentage).

**Table 3:** Baseline laboratory parameters.

Laboratory Parameters		Results	Normal Range
Leukocyte count, mean (±SD)		5824 (±2428) cells/μL	3600–9600 cells/μL
	Leukocytes increase, *n* (%)	4 (8.7)	
Neutrophil count, mean (±SD)		3840 (±1973) cells/μL	1400–6500 cells/μL
	Neutrophils increase, *n* (%)	1 (2.2)	
Lymphocyte count, median (IQR)		1265 (855, 1757) cells/μL	1200–3400 cells/μL
	Lymphocytes decrease, *n* (%)	17 (37.0)	
Hemoglobin, mean (±SD)		13.6 (±1.3) g/dL	12.1–17.2 g/dL
	Anemia, *n* (%)	2 (4.3)	
C-reactive protein, mean (±SD)		14.1 (±3.8) mg/L	0–3.00 mg/L
	Inflammatory syndrome, *n* (%)	26 (60.5)	
AST, mean (±SD)		35.5 (±21.5) U/L	14–36 U/L
	TGP increase, *n* (%)	12 (26.1)	
ALT, mean (±SD)		34.3 (±14.9) U/L	4–35 U/L
	TGO increase, *n* (%)	11 (23.9)	
LDH, median (IQR)		197 (167, 209) U/L	120–246 U/L
	LDH increase, *n* (%)	2 (4.3)	
CK¸ mean (±SD)		69.5 (±19.5) U/L	55–170 U/L
	CK increase, *n* (%)	3 (6.5)	
Urea, median (IQR)		31.5 (20.5, 35.0) mg/dL	19.26–42.8 mg/dL
	Urea increase, *n* (%)	12 (26.1)	
Creatinine, mean (±SD)		0.6 (±0.2) mg/dL	0.66–1.25 mg/dL
	Creatinine increase, *n* (%)	3 (6.5)	
Glucose, mean (±SD)		105 (±14) mg/dL	74–106 mg/dL
	Glucose increase, *n* (%)	16 (34.8)	

ALT—alanine aminotransferase; AST—aspartate aminotransferase; LDH—lactate dehydrogenase; CK—creatine kinase.

## Data Availability

Data is contained within the article.
